# Medical College of Georgia

**Published:** 1876-03

**Authors:** C. A. Evans, Wm. Arnold Adams


### SOUTHERN MEDICAL RECORD.

VOL. VI. MARCH, 1876. No. 3.

### Original Communications.

### MEDICAL COLLEGE OF GEORGIA.

Address by REV. C. A. EVANS, delivered at the Annual Commencement Exercises of the Medical College of Georgia, March 1st, 1876.

*The Ministry of the Medical Scientist.*

The point in speech, or lecture or address, that always gives me trouble is the starting point. I have an antipathy to an introduction, and I think that by the laws of etymology an exordium is *out of order*. There is something wrong about it, or our teachers in the art of discourse would not insist that the first part of the speech shall be written last. In fact, there is a dead level and an intertia in a starting off which are not easily overcome. But beginnings are the puzzles of all philosophers. First truths and facts—those primordials that lie back "in the beginning"—are deeply and darkly sequestered in cavernous concealment too obscure for distinct vision; or else they glow in the primal light too bright for present sight to bear. Yet the world of Mind is now lawfully engaged in looking into the exordium of all this Natural world, which in its own marvelous facts, marshalled in order; in its logic clear as crystal and incisive as diamond; in its diction magnificent and rythmical; is itself a

Divine discourse flowing on in sentences, paragraphs and periods, from an unknown exordium toward no peroration. In this day we have turned our backs on Apocalypse that we may gaze into Genesis, and we do not seem to care so much what will become of the world as to find out how the vast thing began. Is such an inquest on the Past profitless and dangerous? I think it is neither. Is it lawful and safe? I think it is both, provided we are not chained like convicts to its corpse. It may be that Human Quest at this age is rounding a point in the course of its cruise where the final and most favored look may be taken of the shores whence we have sailed, and a true picture be obtained of the origin of things, or the knowledge be lost forever. Mankind has moods, like individual men. It is now in the mood of the Archaist, and should unfetter its spirit and let it drive down deep to the extent of its cable, and go far back to the tightest tension of its tether. For the mood will pass away and may not ever come again. We are ignorant now and will be always so of valuable knowledge because the spirit of certain ages was clipped and caged, and its mood was bound and banished. Now, I say that this earnest interest of the world in *beginnings*, this intense gaze into retreating vistas, these glorious soundings of the deepest seas where worlds of beauty undreampt of hitherto lie, in luscious grace, on couches of such gorgeous drapery as earth's patent plains have not yet produced; this Chinese-like idiosyncrasy of the world's present mood that sends it to stir ancestral ashes and make hoary headed primords prominent. This *pro tempore* disposition to invert the common order of inquiry and retrovert the former way of moving, and to unravel and unwind the wound up ball of Nature, that we might find the beginning of the clew that does seem forever and ever to lengthen as we unwind—all this, I say, is the offspring of a mood Divine, and who fights against it will be found fighting against God. In such a mood discoveries may be made that will brighten Revelation's story. And in such a mood the origin and end of all truth and facts would soon be found, if we but possessed these two attributes—infinity of mental grasp, eternity of mental duration.

I now hope that I have made a start in this discourse, although like Israel in the exodus from Egypt, I have taken my departure *in the dark*. But I do not know that I enter the desert of this discussion with any jewels at all, either the spoils of enemies or the gift of friends. And if in this starting off you have at all suspected the course before us, my strategy has failed. I desired to steal out of port like a blockaded pirate, and get to sea unseen, and cruise without a clearance or a flag. In either case, I am now beyond the blockade, and find myself afloat on the bouyant bosom of *this* delightful theme—*the Ministry of the Medical Scientist.*

It is a comforting reflection that lack of professional qualifications is no hindrance to my cruising in this trackless sea. Free expression of thought is expected in complying with the flattering invitation of this eminent Faculty. The invitation indeed I consider a lawful yet a cunning device to draw out the common views that non-professional men do have of that honorable art of Healing, which occupies a most distinguished place, and fills so large an area in daily popular thought. It is a physician's way of feeling the pulse of the public mind. It is a diagnosis by which the public patient's mental malady or sanity in regard to medical science may be ascertained. And I know full well that, by mere force of professional habit, these skillful scientists are now feeling my pulse, and inspecting my tongue, and looking keenly into the pupil of my eye. I shall be most happy if in the end I am not pronounced unsound.

But in these days of free inquiry and frank speech the humblest subject is permitted to look any kingly science in the face without first donning the attire and wearing the bells of the King's jester. It ought to be the prayer of every science—

"O, wad some power the gifti gie us
To see ourselves as others see us."

I venture then a steady look on you, and go on to discuss my theme—the Ministry of the Medical Scientist—which theme I beg leave to dedicate to you, young gentlemen, who, by authority, begin your professional life to-day.

The terms by which the thought of the theme is expressed were carefully selected. I call the Doctor of Medicine *a scientist*, because he is both a discoverer and a dispenser of valuable knowledge. I define his place in the sciences with the word *medical*, because he employs science in the art of healing. I pronounce his vocation a *ministry*, because it is a service of humanity, whether done in experiment, lectuie or practice. He may, therefore, assume the legend that attaches to a princely office, and inscribe on his official escutcheon the noble words *Ich Lien* —I serve.

I. I made my first observation on this ministry, that it is, by nature, necessarily *conservative*. The first principle of medical science is *Conservation*. To save what is vital force and natural use in human organism is its first care. And its second great principle is *Restoration*. To uplift the prostrate powers of life, and by infusing strength, induce the lagging organs to perform their functions, is its kindly work. On these two great principles this august science seems to be pillared, as the porch of the old Hebrew Temple rested on Jachin and Boaz. They are the prime motives of the thought, feeling and work of the medical scientist. They irradiate his field of study and practice. They pervade his intellectual and emotional constitution, so that to conserve, to restore, become ideas that dominate his life-like laws.

Now, then, it is nearly certain that they whose science is grounded on such principles, and whose thoughts are constantly directed by their force, will be *conservative* thinkers and actors in general. I mean that the necessitated conservatism of medical science tends to making its true disciples conservatives in all respects In the general tenor of their way, amidst the conflict of ultra ideas, they will generally be found in *medias res*. Accordingly, the learned physician is rarely a fanatic. If one exists, he is abnormal—a *lussus medici*, a medical idiosyncrat. A whirly-gig occupies his occiput. Indeed, the medical scientist has the same antipathy to fanaticism as he is said to have to a dose of his own medicine, and for the same reason—both nauseate him. The feud is as old as both antagonists. Esculapius, who is first in historic medical apostleship, became a martyr, the proto-martyr of medical science, and he was slain on the instigation of fanaticism. Legislatures and councils have ostracised the profession, and popular folly has often accused medical men of enjoying the rather questionable luxury of familiar fellowship with the Devil.

It is not surprising, then, in view of the two great principles just named, and the plane of thought on which its disciples move, and the experience of this school, that the medical scientist is the intelligent foe of all sorcery, juggling and humbug. I know that spiteful wit would say that he tolerates no juggling except what he does himself. But this is the wit of the man who is well, and is readily recanted whenever he becomes sick. It is that sort of wit that even a cramp will wither. The medical scientist is genuinely opposed to all pseudo science and all magicians' arts on precisely the same principle that "bears and lions growl and fight"—for "'tis their nature to." Not that it is their nature, *too*, to growl and fight, although they do much of even that among themselves, but it is the nature of their science to repel the unscientific vagaries and the visionary schemes of men. For single example, the physician's views of spiritualism, as having any truth as its basis, or any power as a medicinal agent, are well-known. It is certain that he does not recognize the irregular practice of the disembodied spirits. And to him, and to science generally, all religious teachers may safely remit the treatment of that singular malady.

Yet, with all this conservatism, the scientist under present criticism does not discard discoveries because they are new, as the timid coney shys from the new sheaf of wheat in the field, or flies from the chirp of the first spring cricket. Nor does he cling to ancient error in mere affection for its hoary head, as a bat fastens on the rotting timbers of a venerable turret, because that has been its accustomed roost in the dark. And, on the other hand, he can have no fondness for novelties, and may not, must not, be sensational. He will not sneer at a truth that wears the crown of old age, nor refuse to preserve and ponder a fact that bears the signet of universal and ancient assent. For these two grand sentinel principles that stand at the port of his science, these two guardian principles that guide him across the threshhold of matriculation, into the endless and sublime mysteries that fill his field, forbid the sacrilege of sensational philosophy, and the stupidity of an owlish traditionalism.

II. The protrayal of the Medical Scientist as a Conservative does not preclude this other dilineation, that he is a bold and wise explorer of his broad field, in search of whatever truths and facts may be found there. I do not propose to describe the search of man for "the Truth;" for that was powerfully and beautifully done very recently by a distinguished member of the Medical Faculty. But I desire to follow the medical scientist in his search for what can be found, and see him use what he shall discover. Let me, therefore, make a preparatory general survey of his great field.

There is not always a clear conception had of the difference between *Truths* and *Facts*. The terms are often confounded. But they are not synonymous. *Fact*, in science, is an act, an effect, an event, a thing. But a *Truth*, in science, is a principle or law of a fact, or more than one. Thus it is a discovered *fact* that human blood circulates. But one of *the truths* underlying the fact is that such circulation is necessary to the continuance of human life—and therein lurks a subtle, powerful law. Now, keeping this distinction in view, you understand why the medical scientist is not, in his ministry, a general searcher for "the Truth," but in keen search among the mysteries of his science he must be a particular and an actual gatherer of facts, and a discerner of truths. He must know apparitions from realities, and principles from delusions. His keen eye and delicate touch and sensitive ear must detect Facts that lurk in the little cells, as well as those that loom large and high before him. And of what avail his discovery of facts unless he be a discerner of truths also? He might be skillful in detecting diseases, and yet no healer, because unlearned in their laws.

I state again that this evident distinction between facts and truths leads to an observation of practical importance that there is no fact or principle that can be exalted and distinguished as "the Truth;" nor is there any combination of facts and truths that will produce that fancied thing called "*the Truth.*" Hence the attempt is vain to define truth by a pithy sentence. Neither proverb or maxim has ever replied to that unansewerable question—what is truth? It is the vain question of the restless, the romantic, and the conscience-stricken. It is vain because it proceeds from nothing and leads to nothing. It assumes that, aside from God, there is a thing either concrete or abstract, either actual or ideal, which is "*the Truth.*" And that it is the Truth of which all truths are the progeny, as all rays of the sun are the offspring of its one beating bosom or, that there will hereafter be "the Truth" to which all truths are tending, as some one cavernous sea into which all streams flow.

I admit that in all times and tongues we find this idea of "the Truth," and that it is by universal usage borne on the wings of a figure that is as unreal and impossible as the thing it vainly seeks to signify. There, by that favorite figure of speech, the Truth is described as a focus toward which the luminous lines of all truths converge, as if there were some period past when isolated truths lay almost infinitely apart, and some point in an inconceivable instant of hereafter when all these lines shall meet and there on a quivering mathematical point converge, and blend, and burn, and expire in a final blaze! It was bewildered fancy that formed the figure, and the idea is as fanciful as its symbol. But it is thus that the Truth has been thought of, dreamed of, battled over, and adored as the unknown and unknowable God. It has been searched for with retroverted gaze, as if it were some single element, some primordial fact infinitely insignificant in all respects, save the stupendous one of its supposed infinitely productive power: Or as if it were once some one seed that burst some time, no one knows when nor how, and threw its impregnating pollen over all receptive space, and thus gave perpetuative existence to all that we call Nature and Man: Or, as if it were some word uttered from Eternity's breathing bosom, a ventriloquial sound, a deep drawn sigh of the dark, void, lonely Nothing, and that this sigh moved over the abyss and waked a dead wave, which moved, and in moving lived, and in living brightened and brought forth all the substance and forms of the Facts and Truths in the universe. So, too, "The Truth" is regarded as a thing "to be looked for," as if it were some essence that a future alchemist will extract from all Truths and Facts, when it wlll appear as a drop of crystal dew, to be gazed on for a moment and then be exhaled; or as some blinding blaze, where the faggots of all gathered fruits will be piled in focus around captured and convicted error, and burning down *that*, burn up themselves; or, as if the Truth were some mystic thing like a burning Eye, on whose retina all facts and truths are painted as they successfully arise, until having seen all—having nothing more to see—will wink itself out, and leave the universe as it was in the beginning to begin again, and act over again the same stupendous lie!

Such I conceive are the absurdities of a crude and common notion of "The Truth," as being *e pluribus unum* like this Government of ours; as being one all begetting in the beginning, and all devouring in the end. I find natural science put on this vain search for protoplastic truth in Genesis, and the science of theology seeking its epiphamy in Apocalypse. One delves into the exordium of nature's speech, and the other soars to hear its peroration. But the foundations cannot be discovered nor the altitudes ever surmounted. Let the Doctor of Medicine dream no such dreams. His field is not in the caverns where mummies mould, nor in the clouds where fairies float, but in the actual world, where the facts and laws of disease are such real things as are within the scope of his scientific discovery and use.

I will suggest to you that truths are multitudes, as Facts are many. That Truths are equal, and all Divine, and all eternal. I think they are in a brotherhood, although they do make war on each other as even the tribes of Israel fought. They constitute God's family of Truths and Facts, albeit men will not let them be a happy family. And, therefore, I submit these truer illustrations that truths and facts are leads of treasures that run in parallel lines from an unknown past onward toward eternity beyond, all subject to discovery. They are as parallel bands of distinct colors that blend their edges to form many secondary truths which flow on in concurrent collateral course with their kindred primaries. They seem to form separate systems of spheres, whose lights and glories mingle in the spaces between, while they roll on together toward the endless, as the Universe in its final total motion pours its streams of constellations directly forward. And behind the beginnings of all these harmonious systems of truths and facts stands MIND—stands *He*—Supreme—Personal, filling eternity's background, and there all thought must pause, for of absolute origin man cannot even think. Thus we go back on endless parallels of elements, while beyond us stretch these lines of truth toward no focus where they shall meet to die. There is no sea where they will meet and mingle in a common wave. There is only this broad field of the world where all truths stretch in parallel glory.

Now, such is the field into which the medical scientist is called to make research. What then to him is the practical value of knowing that truths are many, and that they are all discoverable? I will briefly answer. If he start with the idea that there is *one truth*, he will become a medical dreamist. Man, as was so strongly shown in the address referred to, is that unhappy dreamer, and should be so no more. If the medical man take this tangent he will have his visions of one universal solvent of disease. The elixir of life will become the object of his quest. One agent will dance in his daily dreams as the thing that will cure—*or kill.* Some medical monomania will possess him. It may be about the unsophisticated liver that does as well as could be expected. But woe to the patient who has that doctor. It would have been better for that man to have been born without a liver. Or, it may be that he will conceive the truth to be *a sweat*, and, therefore, sweat his patient must, and will, both in person and purse. One idea will become rampant in his mind, and instead of being a medical scientist he will be, if you will pardon me, a medical fool. Paeonitic practice is a delusion, and panaceas are frauds. No Catholicon can be found to ferrit "the ills that flesh is heir to," and drive them away. Elixirs of life elude like the philosopher's stone. Mono-medicatrix is a monomania.

But, we, the patients so-called, the subjects of medical care, cannot afford to have medical men of this type. In witty caricature of pretended medical skill the old couplet makes the pseudo-doctor explain his whole practice briefly in two lines:

> "First I bleeds, and then I sweats 'em,
> And if they die, why then I lets 'em."

This exaggeration is as a distorted shadow thrown upon the wall to excite public laughter, but by it the general feeling is expressed that the human body must not be practiced on by the unlearned, and life must not be trifled with by the unskillful.

We, who are the subjects of medical practice, cannot permit the physician to be other than a medical scientist. Our faith in his skill must be founded on the evidence we have of his science. He may call us his patients by misnomer, but impatient we soon become of him who wears the livery but has not the erudition and the spirit of his profession. He himself is bound by the nature of his vocation to acquaint himself with the mysteries of medical science, and provide himself with the requisites of skillful practice. He must traverse his field with honest diligence in search of all that it will yield. His ministry demands rapid reasoning, close analysis, vivid perception and accurate udgment. It requires obedience of nerve, clearness of vision, subtle hearing and delicacy of touch. Therefore, to be what he is commissioned to become, must he not be a diligent gatherer and gleaner of facts, and a discerner of truths? It is his aim to acquire the erudition that distinguished Thabet Eb'n Abrahim, one example of which will suffice. He acquired such delicacy of touch that by only feeling an artery of his patient a moment he could tell whether he had eaten mutton-chop or beefsteak for breakfast, or drunk cow's milk instead of the milk of the camel for dinner.

Medical Scientists are held bound to explore this field fully to seize and use all facts and truths, because these are within reach of their research.

We cannot, in misplaced charity, accord to them the privilege called liberty of errors in non-essentials, for there can be no non-essentials in this Science. Speculation is for study, experiment for the laboratory, but the clinic must be exact. In theology there is allowable the charming old saying first written in Latin—

In essentials unity,
In non-essentials liberty,
In all things charity.

But in Science, which deals not in forms of words, and which has no ceremony, the fact in everything and truth in the least thing are to be sought and held without concession.

The changeableness of the phenomina of physical and mental maladies also commands him to be alert. Disease is cunning. It is multifold in kinds and symptoms. Like all other error it has a subtle fickleness, and when detected, exposed, and baffled in one attire it will quickly don another. Its combinations are more numerous in variety than the patent locks that bankers fondly use and burglars as fondly pick. And, like the skillful burglar, the Medical Scientist must learn these combinations.

Nor must he suffer himself to be ruled by tradition. After all, traditions are but the relics of thoughts that once lived, breathed and had their uses. They are often an inheritance of chains designed to fetter the inquiring spirit of the after age—mortgages on a devised estate. All traditionary lore must be submitted to tests that try its worth, for in this as in all science "*authority*" must be received with caution and can command assent only when it comes with vouchers in its hands. Precedents in Medical Science are quotable suggestively only. Inflexible formulas are bonds that intelligent and free inquiry must break at will.

Such the eager, honest and cautious quest of him who justly deserves the title which I have given. With patient pursuit as a philosopher, with the cultured eye of an artist, with even the sentiment of a true poet, with the devotion of a lover, and with the philanthropy of the Good Samaritan, he explores his world that he may become the efficient friend of his fellow-man in the hour of need.

III. Submitting these views of this ministry, in regard to its conservative character and its place in science, I will now hold you to a moment's look at the crown which it worthily wears. This crown is its service of suffering man. The medical profession cannot lay down this crown if it would. Mercenary, it cannot be and live. By a law beyond its control, its ministry is public service, and its highest skill must be used gratis to the poor. It is this that makes the science a service that greatens it. And the most skillful are, on that account, most subservient to this law.

There are, in this view of your profession, some peculiarities of which you will have abundant experience. Like Shakspeare's contented swain, you will have to be "glad of other men's good, and content with your own harm." You will have to say amen to the prayer for the sick, although it seems to contravene your prayer for daily bread You will find yourself sometimes in a strait betwixt two feelings, having the humane desire that the people be healthy, and having the conflicting wish for money to soothe your butcher's pains and ease your tailor's aches. You cannot be thankful for a customer like a tradesman, nor be glad at a rise in fever like a broker rejoices in a rise in stocks. You are in this dilemma that if your vocation brings you thrift your friends must suffer, and if they suffer not, why, you must perish.

Is this science then without the deserved recompenses? I answer, that the profession is one in whose practice the acquisition of wealth must be slow but may be sure. In its pursuit the opportunity will rarely rise by which one may spring suddenly into fortune. Happily, the medical scientist may be free from the fond dream of becoming a millionare in a season.

It may even happen that none of these gentlemen who are now beginning their medical career will become afflicted with a plethora of that printed fascination which bears the image and superscription of our Cæsar. I imagine that while I have the honor of addressing young men who will become true medical scientists, that not one expects to be a medical Crœsus. But it is all the nobler in you that you enter a service without the hope of such reward. Such are some of the peculiarities of your position which do themselves train you to a noble manhood in a noble work, where your ministry is exalted. I have placed the healing art abreast, at least, with the foremost sciences, and find all science bringing gifts to its court as kings bring gifts to kings. Perhaps I have even most properly advanced it one step beyond the rest; and if I have, I only imitate the wisest of ancient uninspired sages. When Socrates came to die, his last religious act was sacrifice to deified Esculapius. He turned from all other gods to adore the power that seeks to conserve the *vis vitæ*, and to restore the lost hygiene. His science culminated in the idea that the curative power is man's greatest want. And what if in *that* this Grecian sage felt darkly after Him whom God sent into the world to be the Great Physician! And what if, in this ministry, so much like His own, you shall learn of Him the mysterious secret that neither Galen, Hippocrates, nor Esculapius, nor Socrates knew, but all desired to know: What then, I say? Why, then, there is one more pencil stroke to finish the exalted man whom I have faintly sketched—*he is a Christian medical scientist.* Digging one day in the *debris* of ancient peoples and their tongues, I gained a hint from certain ancient words, and seizing the suggestion, saw, in my mind, an old Phœnician and an old Pelasgian meet four thousand years ago, in the land made famous since as classic Greece. Each had his name for God. In the names they differed widely, but finding that between themselves there was perfect correspondence of ideas, they blent the names in one, and from those blended roots a word in Greek has grown, which we translate "The True." Thus, in the beginning, where haze befogs inquiry, we find this germinal human idea rooting in primal tongues of aboriginal peoples that God is, and that He is *the True;* and toward the Endless, where wearied honest quest folds its pinions to rest upon the heaven builded outer peak of faith, we discover increasing facts and truths which tell us that "*God is, and that He is the True.*" Thus may the various truthful tongues of all the sciences proclaim that there is one God of whom all facts and truths are witnesses, and blend in brotherly confession of *Him*, the Son of God, in whom all truths sustain and every fact conforms.

*Valedictory Address by Dr. William Arnold Adams, of Linton, Ga., a Member of the Graduating Class.*

Ladies and Gentlemen—It is customary. on occasions like the present, to spread rich, ambrosial feasts, where all may taste of delicate viands, and sip from the sparkling goblet filled at Helicon's fount, while showering beautiful wreathes of poesy, gemmed with brilliant pearls of fancy. I will not, however, invite you to partake of such literary dainties, nor need you umbrella yourselves with all your practicability against a shower of poetic metaphors, or a sparkling of classic pearls. I will not ask you to ramble with me through Arno's lovely groves, nor stroll by

"Tempe's classic sunlit streams;"

Nor will we pay a visit to the god's olympus home, to disturb the sciesta of nodding Jove. I rather invite you to a plainly served repast. I hope it may prove palatable, and sincerely pray it may prove beneficial. The times demand, perhaps more than ever, noble men and women in all the vocations of life. Those who will act out noble lives, those who, in life's grand battle fight, will plan with care the campaign sublime, "and having done all, will be able to stand." And then we'll onward move, and moving onward, we'll upward move, to nobler aims—to God.

The times admonish us not to be gazing starward, or, like Thales, we may fall ditchward. The burning rays of misfortune melt the wax wings of imagination. Calliope, herself, accompanied by her music-loving band, would be an intruder in our bankrupt land, and even Orphean notes would discordantly jar with the wailing prayer of our mother South. It is the time for heroism; the opportunity for greatness; and while this is true of all the professions, we need none the less "the true physician," the characteristic of which I will now discuss briefly. My topic means so much that I am at a loss to know what to say or where to begin. I look over this and other countries to find a model; many devotees in our own glorious profession have strewn their pathway with blessings; healing the afflicted, filling thousands of despondent hearts with joy and gladness; turning abodes saddened with disease into joyous homes. We hear the plaudits of appreciating multitudes, whose hearts are aglow with the sense of merit's proved pre-eminence; we see the objects of their applause ever urging their onward course in their path of duty and high calling; regardless of the applause, their ears are inclined to the claims of the afflicted and to the cries of suffering humanity.

We see them self-sacrificing, struggling against pests and epidemics, and shall we select from their number one who shall be worthy of the high name—true physician? Shall he have drank deeply at the fountain of knowledge? Shall all the sciences have adorned his intellect, the richest, lavished by the hands of nature? Who? What man shall constitute the model where so many illustrious examples, by their devotion and self-sacrifice, are enriching the time-honored profession of their choice. Among so many of talents rare, motives pure, and of triumphs varied, we will forbear to select a name, but may we not point to scores as worthy of emulation?

With a heart full of sympathy the true physician labors for those who suffer. In the hour of peril the confiding sufferer sends for the physician with imploring eloquence and heartfelt earnestness begs for assistance, having, it may be, nothing to offer in return save gratitude. Or it may be the anxious heart of a mother, almost wrecked with anguish, in consequence of the suffering of a loved one, whose pleadings start the unbidden tear, and moves his manly soul to action.

"No radiant pearl which crested fortune wears,
No gem that twinkling hangs from beauty's ears,
Nor the bright stars, which night's blue arch adorn,
Nor the rising sun, that gilds the vernal morn,
Shines with such lustre, as the tear that breaks
For other's woe down virtue's manly cheeks."

Character is destiny, is the language of an aphorism, that is a volume of truth, and pregnant with an argument exemplified by history and every-day life illustration. Character is life, and he who would make his life great, must make his character noble. Analyze character, and you analyze life. Know beyond all question the prominent traits of character, and you can prophesy beyond all doubt the salient features of life. Show me a life with no struggles, no defeats, no victories, and I will either show you a character in almost stable equilibrium, or so weak as to be unable to leave the old beaten highway, or too timid to loose the skirts of his father. Some characters are built—they are rather a jumbling together of precious stones and half-burnt brick. Write upon all such the truth that has been echoing over the waste of years: "Unstable as the water, thou shalt not excel."

If our lives be symmetrical and beautiful, symmetrical and beautiful we make them. There is an inner and there is an outer life; the outer is but the reflex of the inner, as it is stamped upon each day-leaf in the record volume. We take our own pictures; at least we are our own life's photographists; good character is the corner-stone to success, the noblest virtue of the true physician.

Another essential element of the true physician, and guarantee of success, is self-knowledge. He who would dispense with it is launching his frail life-bark upon the storm-pregnant deep to drift without chart on the dangerous tide, or to be tossed without compass on the tempest waves. Spectral mists obscures his future, aimless impulses control his present, and humiliating mistakes mark his past. Vainly will he attempt to comprehend life's science, when he has failed to master life's elements, for self-knowledge is the rudiment of universal knowledge, as it is the highest accomplishment of the individual man. The great barrier to the successful prosecution of this study is our own vanity. It is this that prevents us from seeing ourselves "as others see us." Self knowledge ever begets kindness, which is an element of the true physician. Indeed, we too often condemn the erring fallen, when did we know ourselves as we ought we could readily see how, placed under the same circumstances, we too would have yielded. Self-ignorance makes us Pharisees to scorn Publicans.

When you meet an erring brother, oh! do not thank God that you are better, but thank Him that you have never been tempted as he, and giving him the hand of kindness, and speaking words of forgiveness, tenderly lead him back to the right path. When self-knowledge has furnished the bark for the dangerous, haunted voyage of life, when self-control has equipped it, let energy command the oars and unfaultering faith stand at the helm. The true physician recognizes that there is no resting place in the science of medicine. "Dead and uninviting would be that science, the ultimate bounds of whose knowledge had been attained," is the language of a true medical philosopher. The science is progressive; it requires arduous labors, demands great sacrifices, and entails solemn responsibilities.

"Work while it is day." This law is written in all nature; in the purling of the brooklet, as it babbles over its pebbly bed; in the restlessness of the ocean's waves, as they throb over the fathomless deep. In the fretting cascade, and in the twinkling beams of the scintillant starworld; in a word, all nature bows to the decree of God, and shall the true physician, engaged, as he is, in the noblest profession of earth, refuse to to yield fealty to this law? No! Conscious of a moral necessity for "work," and acting in harmony with its promptings, he yields to it a practical recognition. Nor does he esteem pecuniary compensation as the only reward. He has his higher compensations, his hours of triumph. The beacon lights upon the hill top speaks the gratitude of his countrymen, and the caressing smiles of nature penetrate the realms of either, while the tristal battlements of Heaven echo back a welcome.

And in this connection, gentlemen of the Faculty, I must beg leave, ere we bid you a parting adieu, to leave testimony, in behalf of myself and the entire class, to the fidelity and perfection of the lofty ideal of high professional life and duty, which, both by example and precept you have ever kept before our eyes, and impressed upon our minds.

And rest assurd, this added to the patient and persevering assiduity, which you have evolved for our benefit, the deep love of the great science of medicine, and the persistent and unwavering kindness and pains-taking forbearance, which has always marked your intercourse as professors with us as your pupils, has enshrined each and every one of you in our heart of hearts, and gives to the word *farewell*, which we now pronounce, a sadness our bosoms comprehend, but our tongues cannot utter.

And now to you, gentlemen of the graduating class, let me, in conclusion, say a few words: You are on the theshhold of wedding your bride profession, and once wedded, I beg you never be divorced; be true to your profession, and it will be true to you.

Our lives will come to Autumn's hours,
And all may chill and dreary seem;
But even then we'll find some flowers,
And even then some joyous beam.

Repine not, therefore, that thy youth,
And manhood's prime so swiftly flee.
Lo! with advance of years come truth,
New life, new hope, calm joy, for thee.

Unfortunately some men are Mormons in their life's secular wedding; they marry one professon to-day, another to-morrow; some are old "bachelors," and never marry, but drift aimless and purposeless, while many, too many, permit others to select their avocation for life.

Gentlemen, as you have chosen the most honorable profession, let us press forward to the high niche of true physicians. Oh! learn early in your professional career to be unselfish; selfishness is the bane of goodness, the poison of happiness, the murderer of peace, the paralysis of nobility; a delusion; a tantalizing shadow that will deceive your life and work your death. And now, realizing fully the moral necessity for labor; knowing yourselves well, having a right conception of your life, let unselfish love be the motive power. Then devote yourselves, with all your energy, to your life-work. Do your best to acquire fame, power, money; yes, get rich if you can, for nobody has a better right, or a poorer chance, than the "*doctor;*" but in the name of our *Alma Mater*, I entreat you to contend honorably, for if such are to be attained at the sacrifice of honor, let them pass as beneath the dignity of a true physician. Be prudent, kind, temperate in all things. Be refined and gentle in your deportment, with a sympathetic heart and firm character. Above all, let your morals be incorruptible, and your faith in a higher life "be fixed, forever fixed." And when this life grows fainter and fainter, may its fading joys and wasting sorrows die gently away, while memory from the garden of the past will bring sweet flowers and strew them upon your dying pillow; and may the stillness of the twilight hour be thrilled by the enrapturing music of angel bands coming to waft you on their snowy wings to the quiet and peace of Heaven's home, where the melody quavering cycles of eternity ring God's praise.

